# Akabane virus reprograms the PKR‑eIF2α‑SGs/PBs axis to fine-tune cap-snatching efficiency

**DOI:** 10.1080/21505594.2026.2748149

**Published:** 2026-10-12

**Authors:** Zhongyin Liu, Dongjie Chen, Jiamin Qin, Jingjing Wang, Di Wang, Wenke Ruan, Shengkui Xu

**Affiliations:** a Beijing Key Laboratory of Traditional Chinese Veterinary Medicine, College of Veterinary Medicine, Beijing University of Agriculture, Beijing, China; b Institute of Animal Inspection and Quarantine, Chinese Academy of Quality and Inspection & Testing, Beijing, China; c School of Agroforestry and Medicine, The Open University of China, Beijing, China

**Keywords:** AKAV, cap-snatching, SGs, PBs, polysomes

## Abstract

Akabane virus (AKAV) initiates transcription in the cytoplasm via the cap-snatching mechanism. Cytoplasmic host mRNAs act as the primary cap donors, whereas the impact of their subcellular localization on the cap-snatching process remains unclear. Herein, we demonstrate that the AKAV-induced disassembly of polysomes, stress granules (SGs), and processing bodies (PBs) collectively facilitates viral cap-snatching. Mechanistically, AKAV infection activates the PKR-eIF2α signaling axis, leading to the disassembly of polysomes and subsequent translational shutoff. Intriguingly, the viral N protein disassembles SGs and PBs by competing for sequestered RNAs within these granules, whereas mutations of its RNA-binding residues abolish this activity. Moreover, PKR-eIF2α inhibition impairs AKAV replication and cap-snatching efficacy. In contrast, inhibition of SGs via G3BP1 knockdown or chemical agents significantly enhances viral replication and cap-snatching efficiency, indicating that polysomes and SGs disassembly favor caps acquisition. Additionally, N protein-mediated PBs disassembly protects mRNA from degradation, and PBs inhibition enhances cap-snatching. Taken together, AKAV has evolved a sophisticated strategy to reprogram mRNA distribution to create an optimal cellular environment for viral cap-snatching.

## Introduction

*Bunyaviricetes* represent the largest group of RNA viruses, including a wide range of emerging and reemerging pathogens that potentially impact human, plant, and animal health [1,2]. The majority of bunyaviruses are apathogenic in invertebrates, while some cause severe disease in humans and animals, such as Crimean-Congo hemorrhagic fever virus (CCHFV), Hantaan virus (HNTV), Oropouche virus (OROV), and Akabane virus (AKAV) [3]. Currently, there are no effective vaccines or treatments available. AKAV, a member of the genus *Orthobunyavirus* and family *Peribunyaviridae*, mainly causes reproductive disorders in cattle and sheep clinically [4]. Its genome contains three segmented, negative-sense, single-stranded RNA segments: the small (S), medium (M), and large (L). They are tightly encased by nucleoprotein (N) and the large (L) proteins in the virions [5]. AKAV snatches capped-RNA leader sequence from cellular mRNAs to initiate its own mRNA transcription by the cap-snatching mechanism [6]. Thus, AKAV requires abundant cellular mRNAs to complete the lifecycle, posing a key question: how does it efficiently access cellular mRNAs and orchestrate the cap-snatching process?

The subcellular distribution of cytoplasmic mRNAs is tightly linked to their functional states, which can be classified into three distinct categories: “translationally active” (localized to polysomes), “temporarily stored” (sequestered in stress granules, SGs), and “degradation-oriented” (recruited to processing bodies, PBs) [7]. In normally dividing cells, host mRNAs undergo efficient translation, with multiple ribosomes assembling onto individual mRNA molecules to form polyribosomes (polysomes). Notably, polysomes exhibit significantly higher binding affinity for mRNAs compared to other cytosolic RNA-binding proteins (RBPs) [6]. The core trigger for SGs formation is the inhibition of translation initiation [8]. Upon exposure to homeostasis-disrupting stresses such as viral infection, nutrient deprivation, oxidative stress, extreme temperatures, or osmotic changes, cells activate signaling pathways via kinases like double-stranded RNA-dependent protein kinase (PKR), protein kinase R-like endoplasmic reticulum kinase (PERK), and heme-regulated inhibitor kinase (HRI) to phosphorylate eIF2α [9,10]. Phosphorylated eIF2α blocks translation initiation, induces polysome disassembly, and thereby drives the dynamic assembly of untranslated mRNAs with RBPs into SGs [11]. The SGs component Ras GTPase-activating protein-binding protein 1 (G3BP1) functions as a switch triggering RNA-dependent liquid–liquid phase separation (LLPS) and the subsequent SGs assembly [12,13]. The aberrant mRNAs identified by the nonsense-mediated decay (NMD) pathway are transported to the PBs for mRNA degradation [14]. Unlike SGs, PBs form even in unstressed cells. There are multiple enzymes involved in mRNA decapping and degradation in them, which may pose a threat to efficient cap-snatching for cytoplasmic viruses [10,15].

The source of the “caps” utilized by bunyaviruses for their cap-snatching has been extensively investigated for more than a decade. Several bunyaviruses have been reported to target their N proteins to PBs and SGs. Additionally, it has been demonstrated that, beyond PBs, bunyaviruses exploit cytoplasmic mRNAs for the cap-snatching process [16]. However, this study failed to provide solid evidence for a direct correlation between SGs/PBs and the cap-snatching. It seems cost‑effective to snatch caps from PBs for hantaviruses, which are enriched with mRNAs and decapping enzymes, as postulated by Mir et al. [17]. However, the following findings prompt the question: Do PBs serve as a (sole) cap donor source? The rates of cap snatching for Sin Nombre virus (SNV) using premature termination codon (PTC)-containing transcripts are similar in normal and PBs-deficient cells [16]; structural data of hantavirus N protein in the cryo-electron microscopy suggested that no apparent cap-binding site could be observed, rather an RNA-binding domain [6,18]; During cap-snatching, there is no apparent strong preference of cap-snatching for mRNAs either from SGs, PBs, or engaged in translating [16]; Loss of PBs component Dcp2 allows increased Rift Valley fever virus (RVFV) viral transcription, while ectopic expression of Dcp2 impedes viral transcription [19]; The N protein RVFV localizes to PBs and competes with Dcp2, a PBs resident decapping enzyme, for binding to mRNAs, thereby protecting mRNAs from degradation and support the cap-snatching process [19]. In addition, inhibition of SGs assembly via G3BP1 knockdown significantly enhances the replication of Tomato spotted wilt virus (TSWV), indicating that mRNA retention in SGs may hinder virus transcription [16]. Collectively, we propose that cellular mRNAs act as the primary cap donors for AKAV transcription, and their subcellular distribution is critical for this process.

In the present study, we demonstrate that AKAV infection induces cellular translational shutoff via activation of the PKR-eIF2α axis, while the viral N protein disassembles SGs and PBs via compete with them for mRNAs. Finally, the N-mRNA complex protects mRNA from degradation and recruits L protein to initiate viral RNA synthesis. Altogether, AKAV reprograms the PKR -eIF2α-SGs/PBs axis to enhance the efficiency of cap-snatching, thereby promoting viral replication. The detailed results are described below.

## Results

### AKAV infection disassembles SGs in a cell type-independent manner

AKAV requires cellular mRNA caps for transcription initiation. As primary mRNA reservoirs in stressed cells, the temporal dynamics of SGs were analyzed during infection. BHK-21 cells were infected with AKAV, and the assembly of SGs was monitored over time. No visible SGs were detected during the whole course of infection from 12 to 24 hpi (Figure 1(A)), as was the case in BTCs and Vero cells (Figs. S1(A), S1(C)).

**Figure 1.**
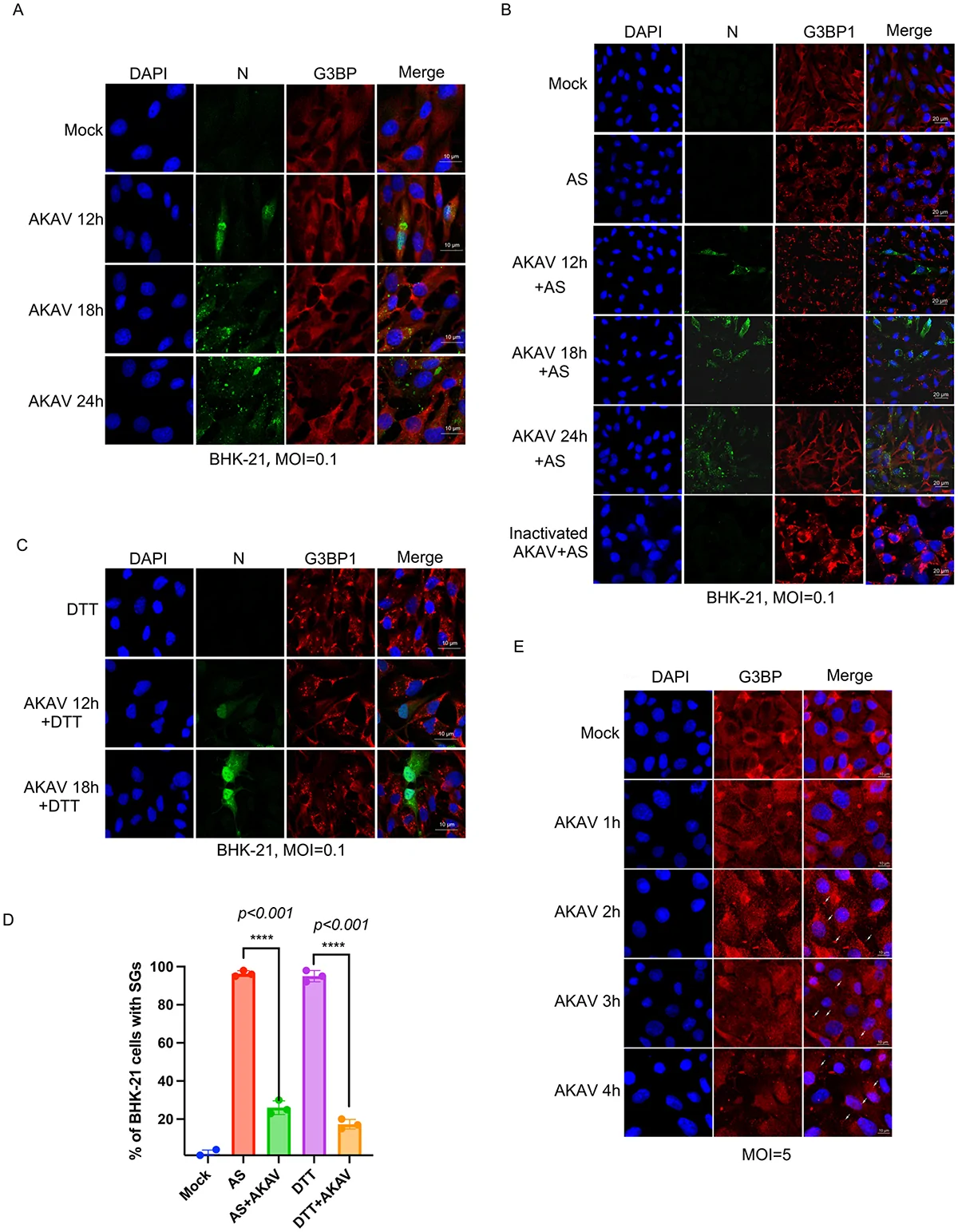
AKAV infection induces SGs disassembly.

(A) BHK-21 cells were infected with AKAV. At 12, 18, and 24 hpi, the cells were subjected to IFA. The cell nuclei were stained with DAPI (blue). Antibody against the N protein (green) was employed to detect AKAV infection. Antibody against G3BP (red) served to label SGs. Scale bar = 10 μm. (B, C) BHK-21 cells were infected with AKAV. The kinetics of SGs assembly were determined at indicated time points. AS (Sodium arsenite) (0.5 mM for 30 min) and DTT (2 μM for 1 h) were used as SGs inducers. (D) The statistical analysis of SGs-positive cells in BHK-21 cells was conducted. The counting was performed in 3 random fields for each of the AKAV-infected for 24 h-, AS‑ or DTT-induced, or Mock-mock‑infected groups. The data were presented as mean ± SD at least three independent experiments (t-test, *indicates *p* < 0.01). (E) BHK-21 cells were infected with AKAV. At 1, 2, 3, and 4 hpi, the kinetics of SGs assembly were determined at indicated time points.

To further verify if AKAV infection disrupts SGs actively, two SGs inducers were employed: sodium arsenite (AS, an HRI activator) and DL-dithiothreitol (DTT, a PERK activator). Surprisingly, the formation of SGs was markedly suppressed in AKAV-infected BHK-21 cells compared with that in uninfected cells when treated with SGs inducers (Figure 1(B–D), S2D). The same phenomenon was also observed in both Vero and BTC cell lines (Figs. S1 (B), S1 (D), S1 (E)). Notably, SG assembly was observed at early phase of infection (Figure 1(E)), as was phosphorylation of PKR and eIF2α (Fig. S2 (E)). Nevertheless, UV-inactivated virus lacked the capacity to trigger this process (Figure 1(B)). This indicates that AKAV infection actively inhibits SGs assembly in a cell type-independent manner.

### AKAV infection triggers SGs upstream PKR-eIF2α signaling axis and host shutoff

Viruses can subvert SGs formation via multiple strategies [20,21] (Figure 2(A)). eIF2α is the central trigger for SGs assembly, thus, its phosphorylation was first investigated. The results showed that eIF2α phosphorylation levels gradually increased as the infection advanced (Figure 2(C), S2 (A)). Notably, while AKAV inhibited AS-induced SGs assembly, it did not suppress AS-triggered eIF2α phosphorylation (Figure 2(B)), indicating that AKAV might inhibit SGs formation by directly targeting SGs components. G3BP1, a key switch for SGs assembly, is a valuable target for viral hijacking [20]. Surprisingly, its expression and integrity remained unaltered in both BHK-21 and Vero cells throughout the entire course of infection (Figure 2(D), S2 (B)), implying that AKAV has evolved a new strategy to modulate SGs assembly.

**Figure 2.**
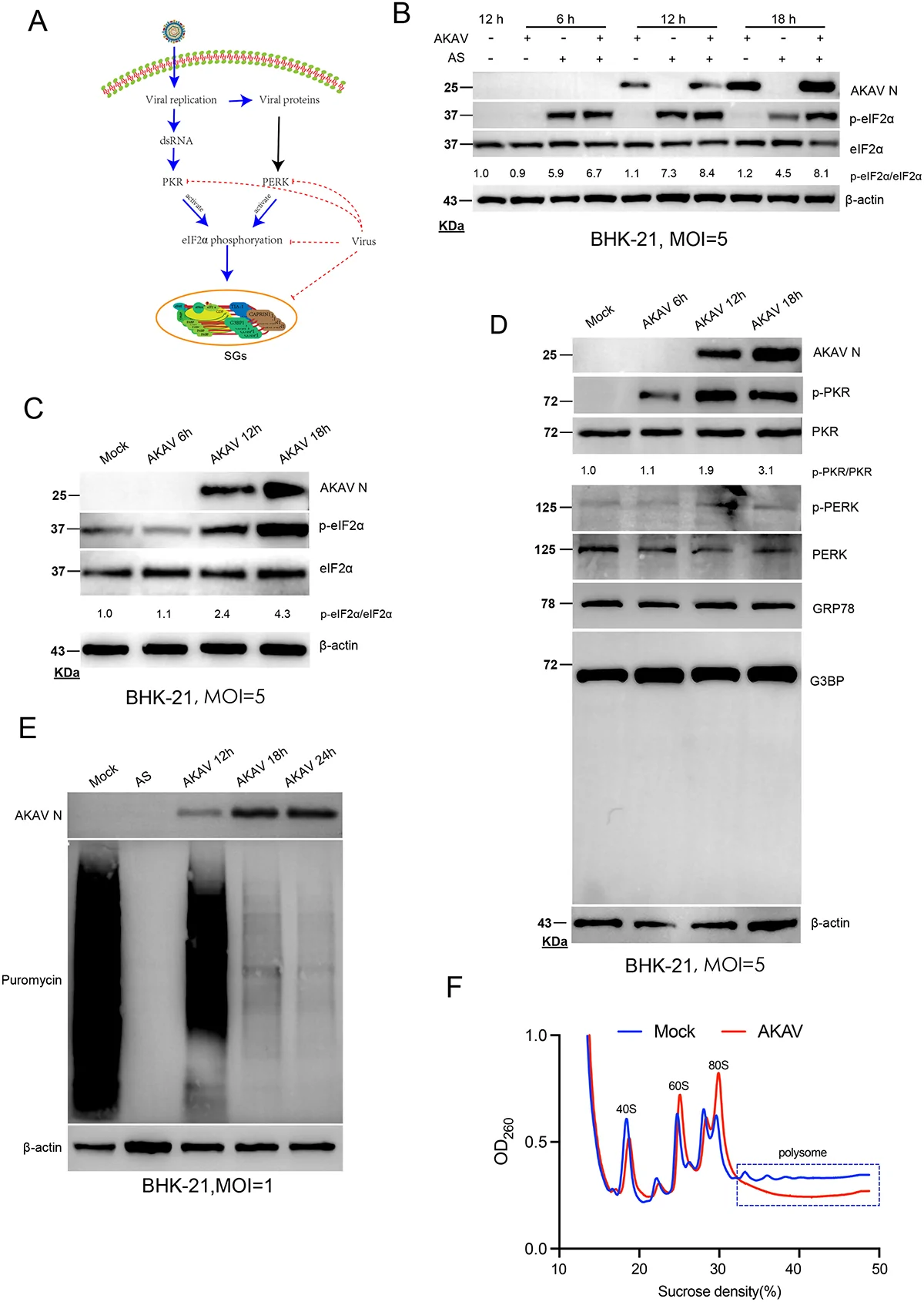
AKAV infection triggers the phosphorylation of eIF2α via PKR.

(A) A schematic diagram illustrating the formation of SGs induced by viral infection. (B) BHK-21 cells were infected with AKAV at an MOI of 5. The cells were harvested at indicated times for Western blot analysis to detect eIF2α and p-eIF2α. β-actin served as a loading control, and N protein served as an indicator of infection. AS acted as an activator of eIF2α phosphorylation. (C) The expression and phosphorylation levels of eIF2α in BHK-21 cells were analyzed by Western blotting during AKAV infection. (D) The activation of PKR, PERK, and the G3BP1 status were detected by Western blotting in AKAV-infected BHK-21 cells. (E) BHK-21 cells were infected with AKAV at an MOI of 1. Subsequently, the infected cells were treated with 10 μg/mL puromycin for 25 min and harvested at various timepoints. The whole‑cell lysate was then subjected to Western blot analysis with an antibody against puromycin. (F) BHK-21 cells were infected with AKAV (MOI = 5) for 12 h, and the whole cells were collected and subjected to polysome profiling.

The eIF2α kinases PERK and PKR are predominantly involved in virus infection. To clarify the upstream kinases responsible for eIF2α phosphorylation, PERK, and PKR phosphorylation were detected. We showed that the expression and phosphorylation of PERK were not altered in AKAV-infected cells (Figure 2(D)). Consistently, the expression of glucose-regulated protein 78 (GRP78), the key indicator of endoplasmic reticulum stress, also remained unaltered (Figure 2(D)). In contrast, PKR was significantly phosphorylated in AKAV-infected BHK-21 and Vero cells (Figure 2(D), S2 (B)), indicating that eIF2α is phosphorylated by PKR. eIF2α phosphorylation is closely correlated with the stability of polysomes and cellular translation efficacy. To explore whether AKAV infection results in host translation shutoff, puromycin, which can be randomly incorporated into nascent polypeptides, was employed to monitor the translation efficacy of host cells [22]. BHK-21 cells infected with AKAV displayed translation shutoff at 12 hpi, and grew progressively more pronounced as the infection advanced (Figure 2(E)). In addition, IFA results also proved that AKAV infection inhibits host cell translation at the single-cell level (Figures S2 (C)). Polysomes are hallmark complexes of actively translating mRNAs, and polysome profiling demonstrated that AKAV infection elevated 80S monosome levels while reducing polysome formation (Figure 2(F)). Collectively, AKAV activates the PKR-eIF2α axis to trigger cellular shutoff.

### AKAV N protein suppresses SGs assembly via its RNA-binding domain

AKAV encodes four structural proteins and two non-structural proteins. To screen for viral proteins responsible for SGs inhibition, BHK-21 cells were transfected with plasmids expressing HA-tagged viral proteins in the presence of AS. AS was unable to induce SGs assembly in N-expressing cells, while it efficiently promoted SGs formation in cells expressing Gn, Gc, L, Nss, or Nsm (Figure 3(A)), indicating that the N protein alone is sufficient to inhibit SGs assembly.

**Figure 3.**
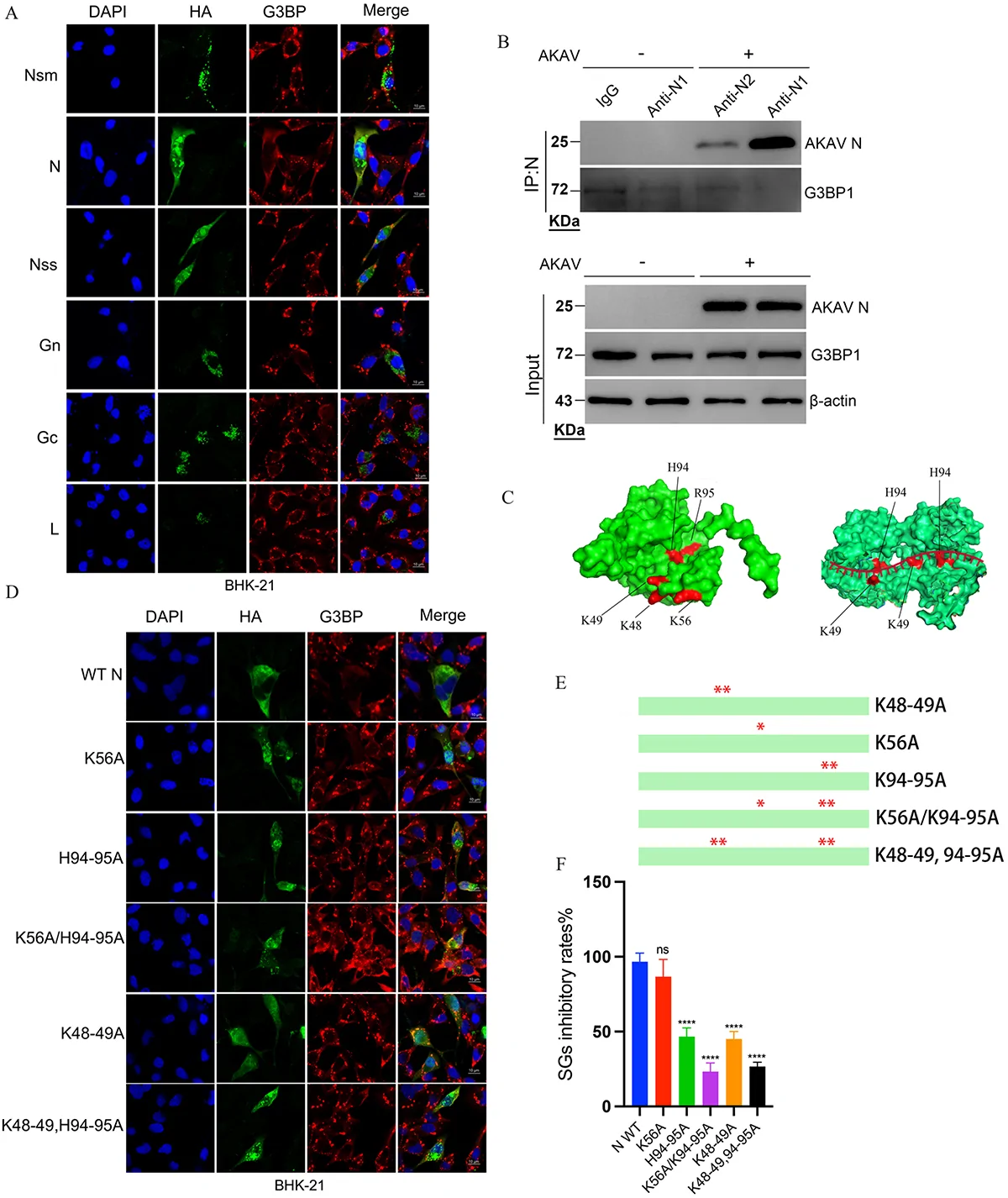
The N protein is responsible for the blocking of SGs.

(A) BHK-21 cells were transfected with plasmids encoding HA-tagged viral proteins for 36 h. Prior to fixation, the cells were treated with AS at a final concentration of 500 μM for 30 min. IFA was carried out using antibodies against HA and G3BP. (B) BHK-21 cells were infected with AKAV at an MOI of 5 for 24 h. The cell lysates were collected and co-immunoprecipitated with two different antibodies against N. The immunoprecipitants and the whole-cell lysates were subjected to Western blot analysis. (C) The structure of the N monomer (left) and tetramer (right, half presented) are predicted by AlphaFold3 (https://alphafoldserver.com/about) and the amino acids K56, K48, K49, H94, and R95 are highlighted in red. (D, E) The inhibitory effect of SGs in BHK-21 cells upon ectopic expression of wild-type N and its mutants. (F) The inhibitory rates of SGs in BHK-21 cells upon ectopic expression of wild-type N and mutants.

To gain an in-depth understanding of the mechanism by which the N protein inhibits SGs formation, Co-IP assays were employed to probe the association between the N protein and G3BP1, a central node protein in SGs assembly. However, there was no apparent G3BP1 co-precipitated with N by anti-N mAb (Figure 3(B)). Additionally, N protein exhibited a distinct distribution pattern compared with cellular G3BP1 in both N-overexpressing and AKAV-infected cells (Figure 3(A),1(A)). Thus, the N protein inhibits SGs not via targeting G3BP1.

The N protein binds to viral RNAs in virions with its RNA-binding activity mediated by the amino acid residues K56, H95, and H96 [23], which are highly conserved among the *Orthobunyavirus genus* (Fig. S3 (A), indicated by asterisk in red). We mutated the amino acids to alanine one by one or simultaneously (Figure 3(C,E)). The results showed that the N protein mutants K94-95A and K48-49A failed to inhibit SGs formation, whereas mutant K56A-located outside the RNA-binding groove-did not exhibit such an effect (Figure 3(D,F)). Furthermore, we also found that ectopic expression of the N protein did not affect eIF2α phosphorylation (Fig. S3 (B)). Taken together, the N protein may disassemble SGs via its RNA-binding domain.

### The N protein competes with G3BP1 for mRNA binding

We hypothesize that AKAV induces SGs disassembly by binding to sequestered mRNAs within the granules. To verify this hypothesis, an oligo-(dT)-RIP assay was conducted to assess N/G3BP1–mRNA interactions. The results showed that mRNAs were successfully precipitated by oligo(dT) (Figure 4(A)). Then, Western blot was applied to detect the components of the precipitated mRNA complex. Under unstressed conditions, endogenous G3BP1 exhibits low-level binding to mRNA, and this interaction is significantly enhanced following AS treatment (Figure 4(B)). However, in AKAV-infected cells, this interaction is markedly abolished; instead, the N protein is precipitated by oligo-(dT)-conjugated magnetic beads (Figure 4(B)), indicating that N protein competes with G3BP1 for mRNA and displaces them from the complex. Furthermore, the competition for mRNA binding between the viral N protein and G3BP1 was examined *in vitro*. Incubation of purified viral N protein with AS-treated cells disrupts the interaction between G3BP1 and mRNA (Figure 4(C)).

**Figure 4.**
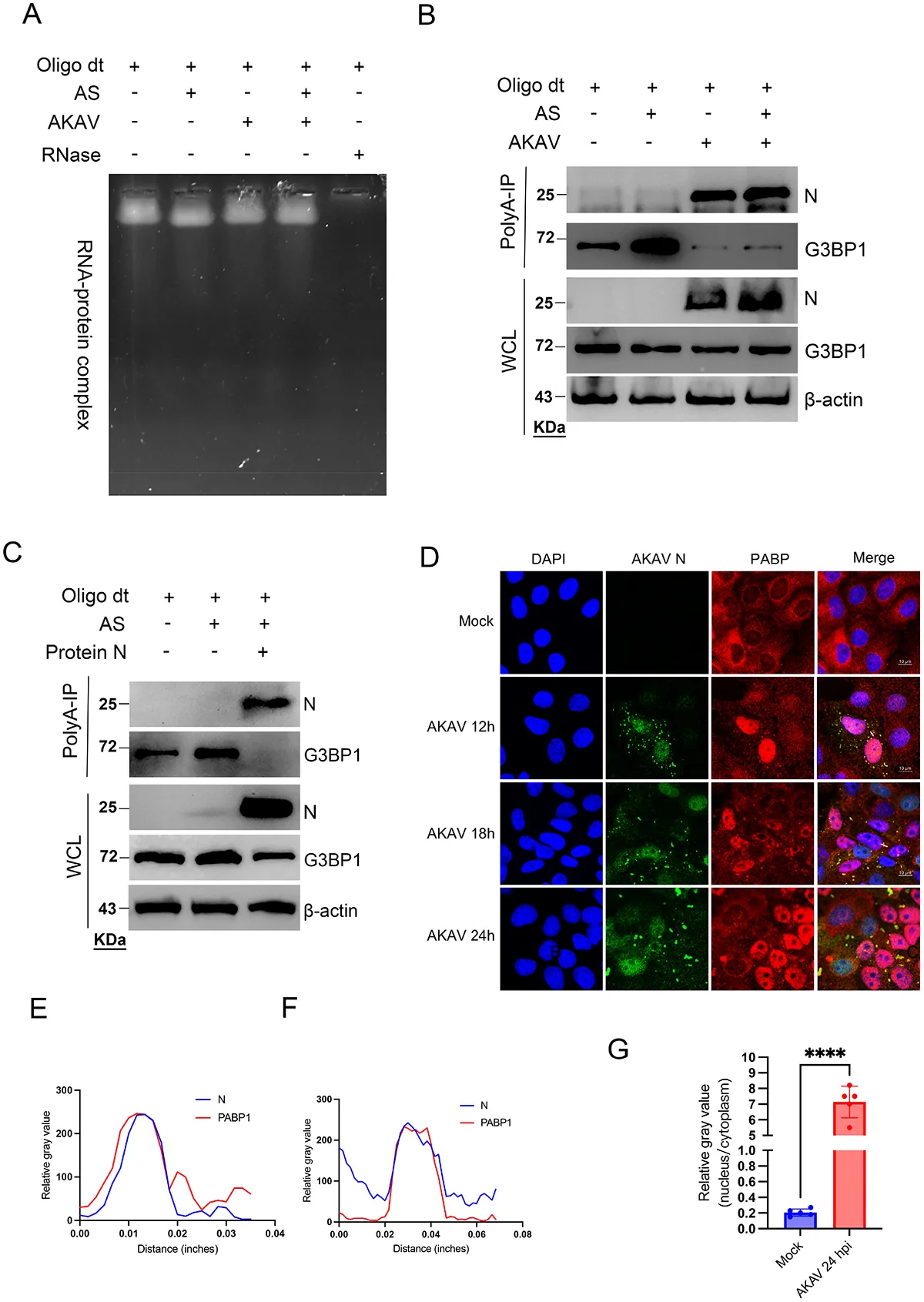
N protein disturbs the interaction between mRNA and G3BP1.

(A) Oligo-dT pull-down was conducted on BHK-21 cells treated with AS (500 μM; 1 h). The RNA was visualized by SYBR Gold staining. (B) Western blot analysis was carried out to detect the protein in the oligo-dT-precipitated lysate using antibodies specific to G3BP1 and N. (C) Purified N protein was co-incubated with oligo-dT-precipitated lysate for 2 h at 4 °C, then Western blot analysis was performed to detect the levels of N and G3BP1. (D) The distribution of N protein and poly(A)-containing RNA. Vero cells were infected with AKAV at an MOI of 1. Then IFA was carried out to visualize the localization of N and PABP1. (E, F) The fluorescence intensity profiles of N (blue) and PABP1 (red) were measured along the line drawn on the panel by ImageJ software at 12 hpi and 18 hpi. (G) Quantification of the relative gray value of PABP1 in the nucleus and cytoplasm of BHK-21 cells at 24 hpi compared with mock-infected cells. The **** indicate a highly significant difference (*p* < 0.0001).

Poly(A)-binding protein 1 (PABP1) associates with actively translating mRNAs on polysomes. To investigate whether the N protein interacts with cellular mRNAs in the cytosol, IFA was performed to examine the localization of N and PABP1. The results demonstrated that N protein colocalized with PABP1 during the early stage of virus infection (Figure 4(D-F)). As the infection progressed, most PABP1 was redistributed and retained in the nucleus, whereas the remaining cytoplasmic PABP1 colocalized with N protein (Figure 4(F,G)). The colocalization between the N protein and poly(A)-containing mRNAs was also detected by IFA. The results revealed that the N protein partially colocalized with poly(A)-containing mRNAs in AKAV-infected cells (Fig. S4). Taken together, the N protein disrupts the G3BP1–mRNA complex and disassembles SGs.

### PKR inhibition hinders virus replication by attenuating cap-snatching

Generally, activation of PKR, an antiviral protein, hinders virus replication by halting protein synthesis [24]. The PKR activation inhibitor, PKR-IN-C16, was employed to investigate the impact of PKR phosphorylation on AKAV replication. Unexpectedly, following PKR-IN-C16 treatment, the expression level of the N protein was substantially lower than that in the untreated group (Figure 5(A)). Similarly, the viral titers in BHK-21 cells were markedly reduced when treated with PKR-IN-C16 (Figure 5(B)), indicating that AKAV-induced PKR activation promotes its replication.

**Figure 5.**
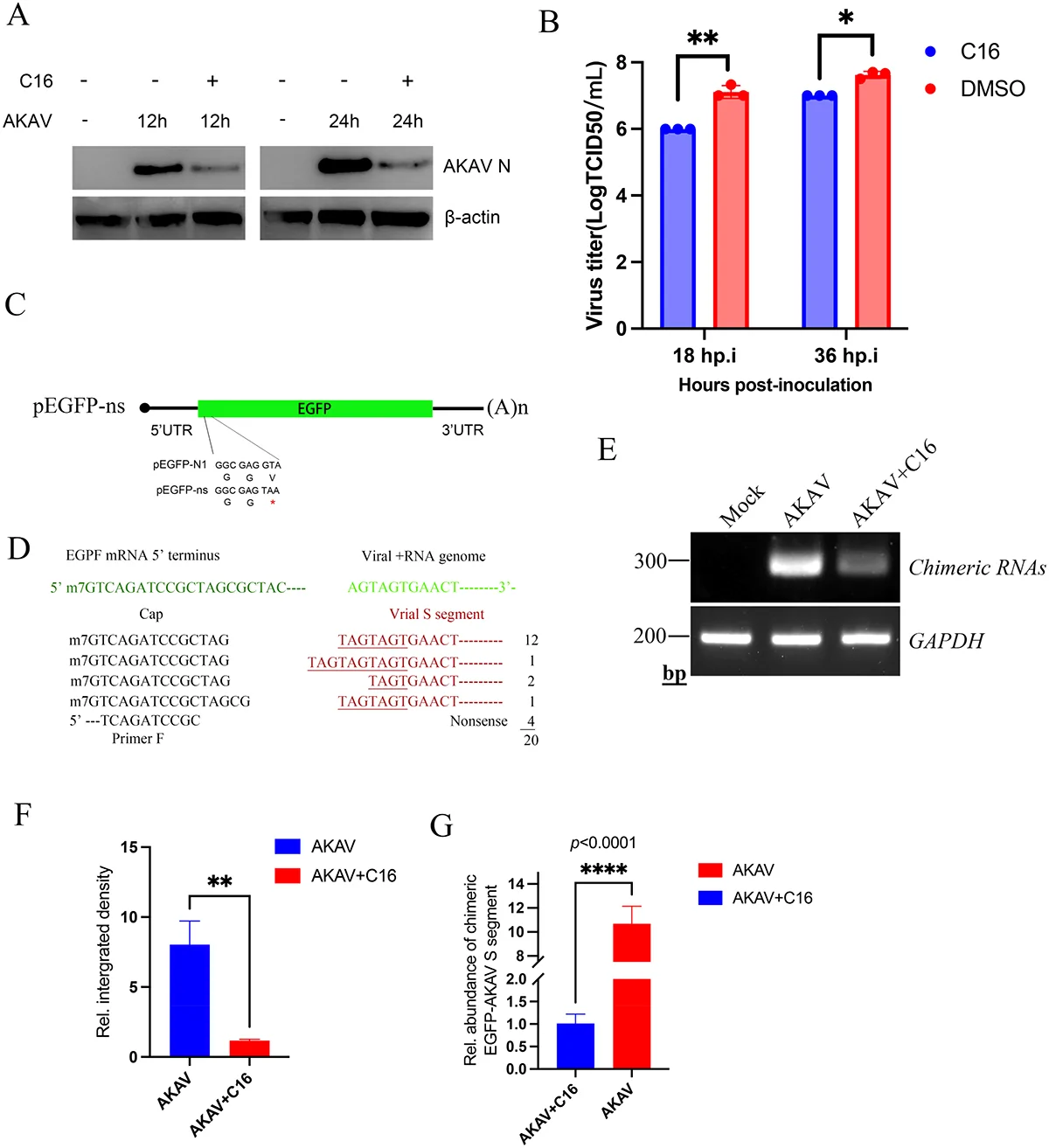
PKR inhibition hinders AKAV cap-snatching efficacy.

(A) The expression levels of N protein in AKAV-infected cells at 12 hpi and 24 hpi were detected by western blotting assay after treatment with PKR-IN-C16 or DMSO. (B) The viral titers in cells treated with PKR-IN-C16 and DMSO were detected by the TCID_50_ method at 18 hpi and 36 hpi. (C) A schematic diagram is presented to illustrate the construction of the plasmid pEGFP-ns, which is designed for transcribing nonsense mRNAs and used as “cap donors.” (D) The cap sequences are depicted in blue, and the viral S segment sequences are depicted in green. Sequence analysis of chimeric RNAs derived from EGFP nsRNA and viral S segment. The chimeric RNAs were detected using a sense primer that matches the 5’ end of the GFP RNA and an antisense primer complementary to the AKAV S segment mRNA. RT-PCR products were cloned into the pEASY Blunt Zero vector, and 20 colony forming units were randomly sequenced. (E) Equal amounts of cDNA were used as templates for the amplification of chimeric RNAs. GAPDH was used as loading control. (F) Statistical analysis of the gray value of the band corresponding to the chimeric RNAs was performed using ImageJ software. (G) Relative abundance of chimeric RNAs was determined by qRT-PCR. (*, *p* < 0.05; ***, *p* < 0.001).

Cellular mRNAs harboring a premature translation termination codon (PTC) serve as preferred “cap donors” during bunyavirus transcription [17]. Thus, we transfected the constructed plasmid pEGFP-ns into cells to provide caps, and its feasibility was verified by PCR with different primers (Figure 5(C), S5). The Cap-N-F1/Cap-S-R primer set was used for further research. Activation of the PKR-eIF2α axis promotes polysome disassembly and release of mRNA. To test whether the PKR-IN-C16 affects the cap-snatching process, RT-PCR and qRT-PCR were applied to assess the cap-snatching efficacy. The results showed that, compared with the DMSO-treated group, caps derived from pEGFP-ns were significantly lower in viral mRNAs in the PKR-IN-C16-treated group (Figure 5(E-G)). Additionally, the PCR product was cloned into the pEASY-Blunt Zero vector and sequenced by Sanger sequencing (Figure 5(D)). Together, PKR phosphorylation may promote AKAV replication by enhancing cap-snatching efficacy.

### SGs inhibition promotes AKAV replication

AKAV induces the formation of SGs at the early stage of infection, whereas the viral N protein mediates SG disassembly as the infection progresses. To investigate the impact of SGs on AKAV replication, we inhibited SG formation with chemicals and siRNAs. PT129 blocked SG assembly via targeting G3BP1, and this treatment enhanced viral replication, protein expression, and cap-snatching (Figure 6(A-C), S3C), indicating SGs impair AKAV cap-snatching and propagation. As a critical switch for SG assembly, G3BP1 knockdown notably upregulated viral N protein expression detected by Western blot (Figure 6(D)). Moreover, G3BP1 silencing significantly elevated viral titers and cap-snatching efficiency (Figure 6(E,F)). Taken together, AKAV-induced SG disassembly enhances AKAV’s cap-snatching activity and propagation.

**Figure 6.**
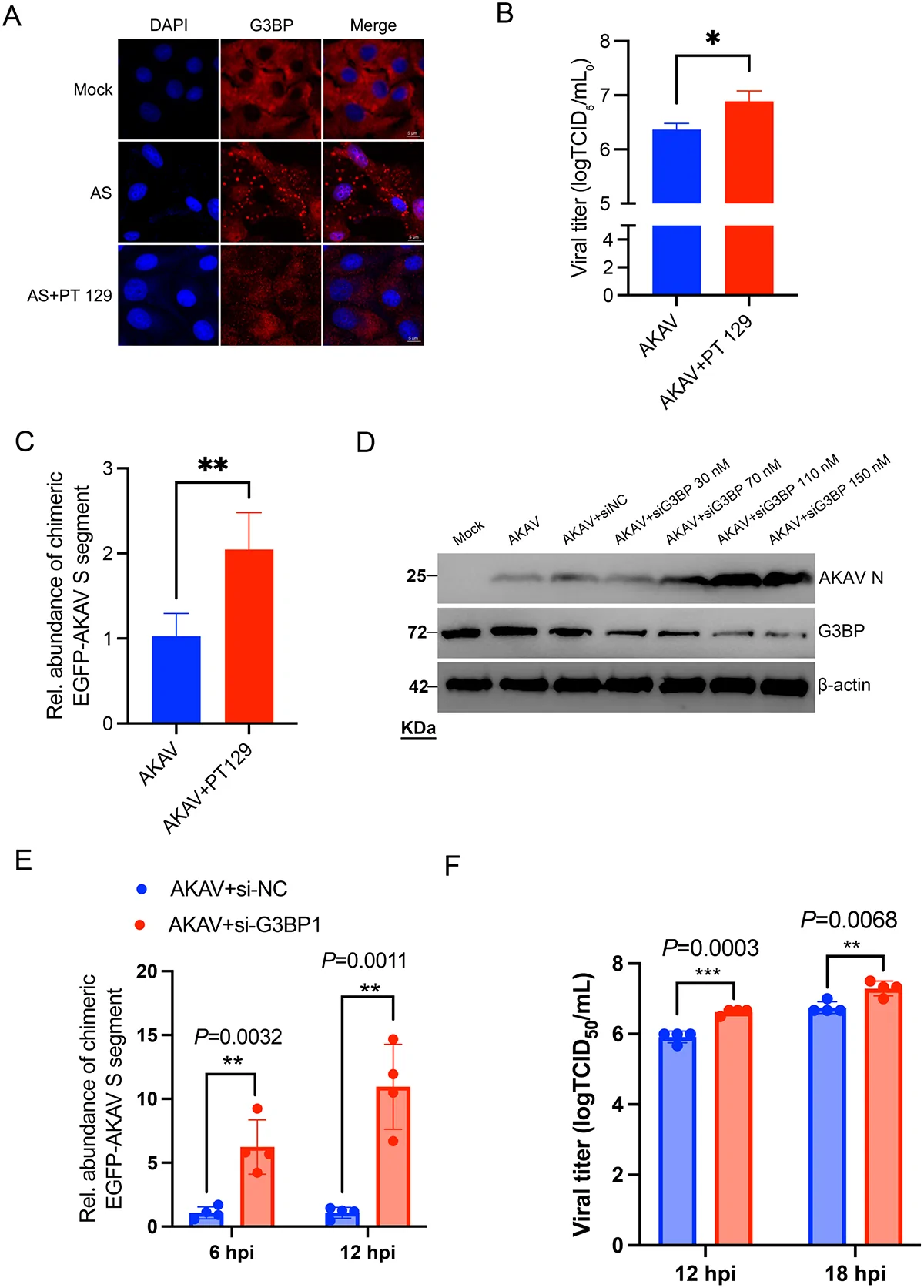
SGs inhibition promotes AKAV replication.

(A) BHK-21 cells were pretreated with the SG inhibitor PT-129 (5 μM) for 12 h, followed by AS treatment (0.5 mM) for 30 min prior to fixation. (B) BHK-21 cells were infected with AKAV in the presence of PT-129 (5 μM) and incubated for 12 h, followed by viral titer determination via the TCID_50_ method. (C) BHK-21 cells were transfected with plasmid pEGFP-ns 24 h prior to AKAV infection (MOI = 5) in the presence of PT-129 (5 μM), followed by 12 h incubation. The chimeric RNA was determined by qRT-PCR (**, *p* < 0.01). (D) BHK-21 cells were transfected with two siRNAs targeting G3BP1 for 36 h to knock down G3BP1 expression. Then, the cells were infected with AKAV for 12 h. The expression of G3BP and N protein was detected by Western blotting. (E) BHK-21 cells were cotransfected with siG3BP siRNA and pEGFP-ns for 36 h, followed by AKAV infection for 12 h. The chimeric RNAs were determined by qRT-PCR at 6 and 12 hpi. (F) The BHK-21 cells were treated with siG3BP (100 nM) for 36 h, followed by AKAV inoculation. Then the viral titers were detected by the TCID50 method at 12 and 18 hpi (*, *p* < 0.05; **, *p* < 0.01; ***, *p* < 0.001).

### AKAV infection disassembles PBs via the RNA-binding domain

AKAV inhibits SGs by competing for mRNAs, which are shared by many other RNA granules. PBs represent another major class of membraneless cytoplasmic mRNP condensates that govern mRNA turnover and translational repression. Thus, their assembly was also determined. Surprisingly, we found that the PBs were also dispersed in the AKAV-infected cells (Figure 7(A)). Notably, the N protein formed foci in virus-infected cells. At 12 hpi, these foci were associated with – or rather, docked to – several PBs, whereas this colocalization disappeared by 24 hpi (Figure 7(A-C)). Meanwhile, several docking PBs showed dispersed patterns or became much smaller compared with those in mock-infected cells (Figure 7(A,E)). Similar findings were obtained in BHK-21 cells (Figure 7(D,F)). Thus, we hold that N protein forms foci during infection, which are associated with PB redistribution and may contribute to PB disassembly.

**Figure 7.**
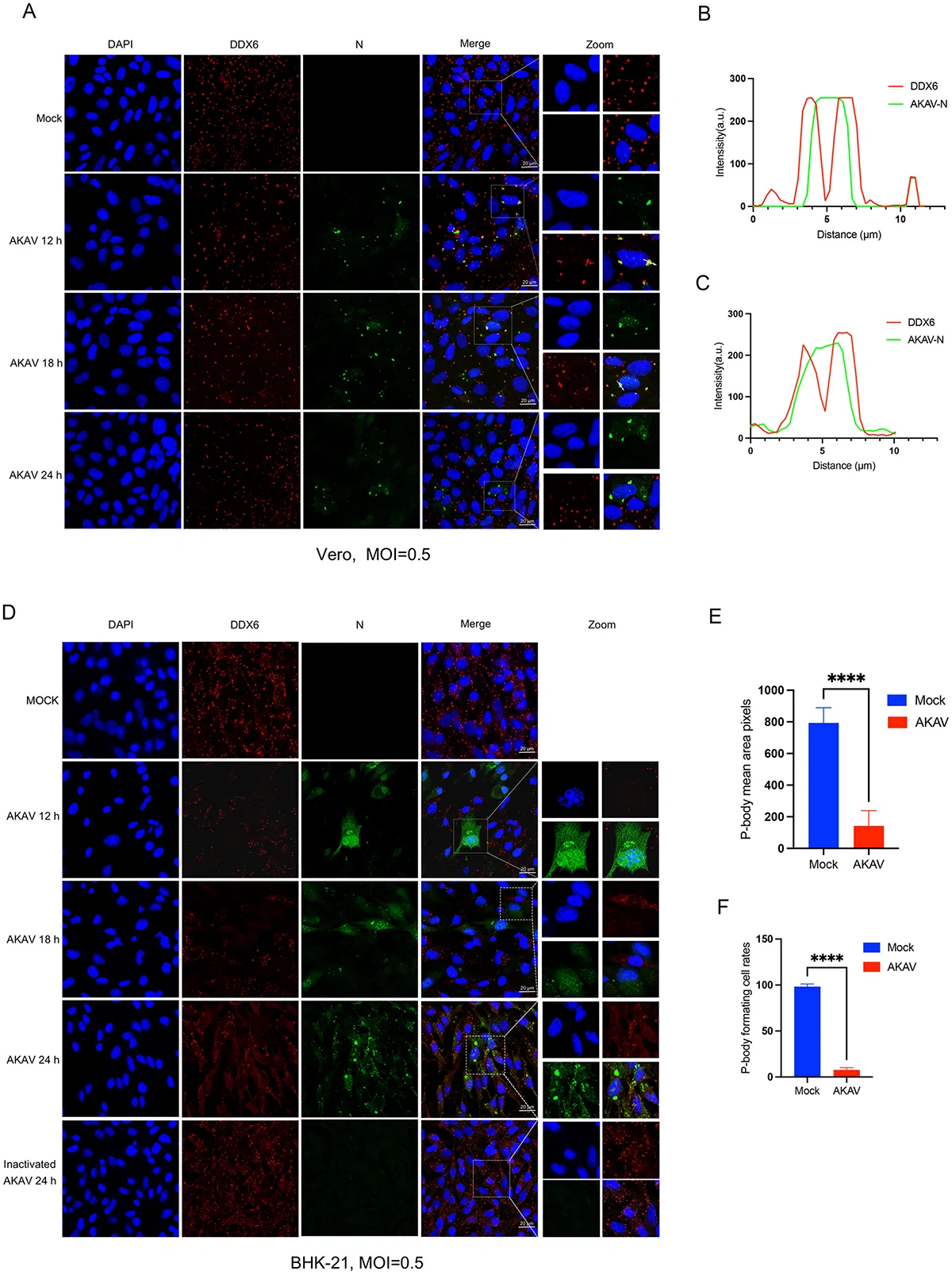
AKAV infection blocks PBs assembly.

(A) Vero cells were infected with AKAV at an MOI of 1 and fixed at different time points. The cells were immunostained with an antibody against DDX6 (a marker for PBs) and an antibody against the viral N protein. (B, C) The fluorescence intensity profiles of N (green) and DDX6 (red) were measured along the line drawn on the panel by ImageJ software at 12 hpi and 18 hpi. (D) BHK-21 cells were infected with AKAV at an MOI of 0.5. At various time points, the infected cells were subjected to IFA to observe the formation of PBs. (E) Statistical analysis was conducted on the mean area (in pixels) of PBs in both mock-infected and AKAV-infected cells at 24 hpi. (F) Statistical analysis was carried out on the rates of cells in which PBs formed in both mock-infected and AKAV-infected cell groups at 24 hpi.

To screen for viral proteins responsible for PBs inhibition during AKAV infection, BHK-21 cells were transfected with plasmids expressing HA-tagged viral proteins. The results indicated that the PBs were disassembled only in N-expressing cells, suggesting that the N protein uniquely impacted PBs structure (Figure 8(A)). Similarly, N also blocks PBs assembly in Vero cells (Fig. S6). To gain deeper insights into the mechanism through which the N protein curtails PBs formation by directly interacting with PBs constituents, Co-IP assay was employed to study the association between the N protein and DDX6, a central node protein of PBs. However, no apparent DDX6 was co-precipitated with N by anti-N mAb (Figure 8(B)). In addition, the N protein exhibited a distinct distribution pattern compared with cellular DDX6 in AKAV-infected cells, although PBs were found to dock on N foci (Figure 7(A,D)). To test if the RNA-binding ability of the N protein affects anti-PBs function, the PBs assembly was observed in N protein mutant-expressing cells. This indicates that, similar to the mechanism underlying N protein-mediated SG inhibition, N protein suppresses PBs not via direct binding to PB constituent proteins but through its RNA-binding capacity. Our results demonstrated that mutation of the RNA-binding domain in the N protein abrogated its anti-PBs activity (Figure 8(C,D)).

**Figure 8.**
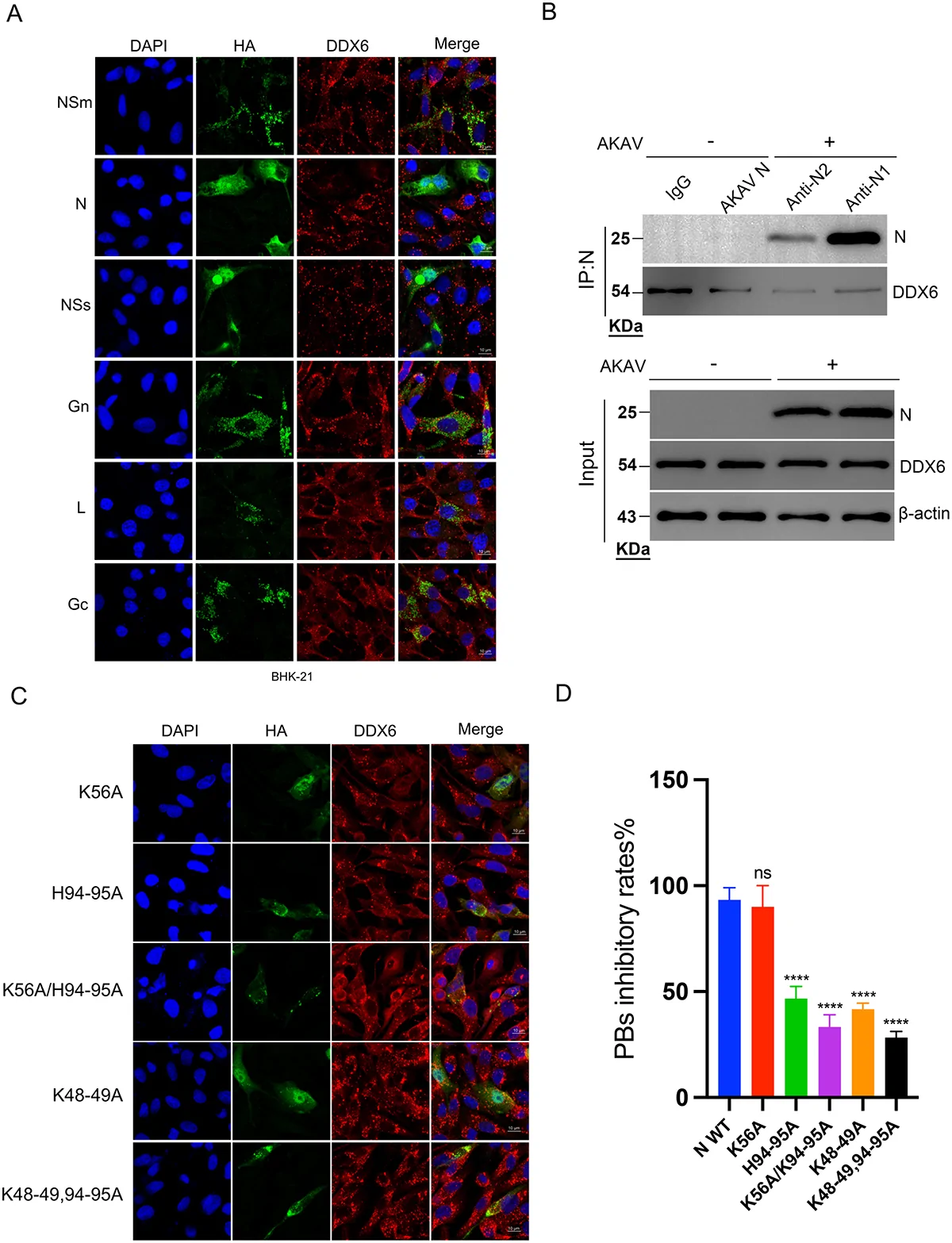
N protein disturbs PBs formation.

(A) BHK-21 cells were transfected with plasmids encoding HA-tagged viral proteins for 36 h. Subsequently, the cells were fixed, and IFA was carried out using antibodies against HA and DDX6. (B) BHK-21 cells were infected with AKAV at a MOI of 5 for 24 h. The cell lysates were collected and co-immunoprecipitated with an antibody against N. The immunoprecipitants and the whole-cell lysates were subjected to Western blot analysis. (C) Ectopic expression of N protein mutants in BHK-21 cells was assessed for effects on PB formation. (D) The PBs inhibitory rates in N protein‑mutant‑expressing cells.

### N-mediated PBs inhibition prevents mRNA degradation and facilitates cap-snatching

PBs are the primary degradation sites for mRNAs. We therefore investigated the effects of AKAV N protein-mediated PBs inhibition on mRNA abundance. Results showed that the mRNA level was approximately 2-fold higher in N-overexpressing cells than in cells transfected with the empty vector pEGFP-N1, indicating that N protein-mediated PBs inhibition protects mRNA from degradation (Figure 9(A,B)). Dcp2, the catalytic component of the mRNA decapping machinery, is involved in RNA degradation. Loss of Dcp2 promotes AKAV replication and the cap-snatching efficacy (Figure 9(C-E)).

**Figure 9.**
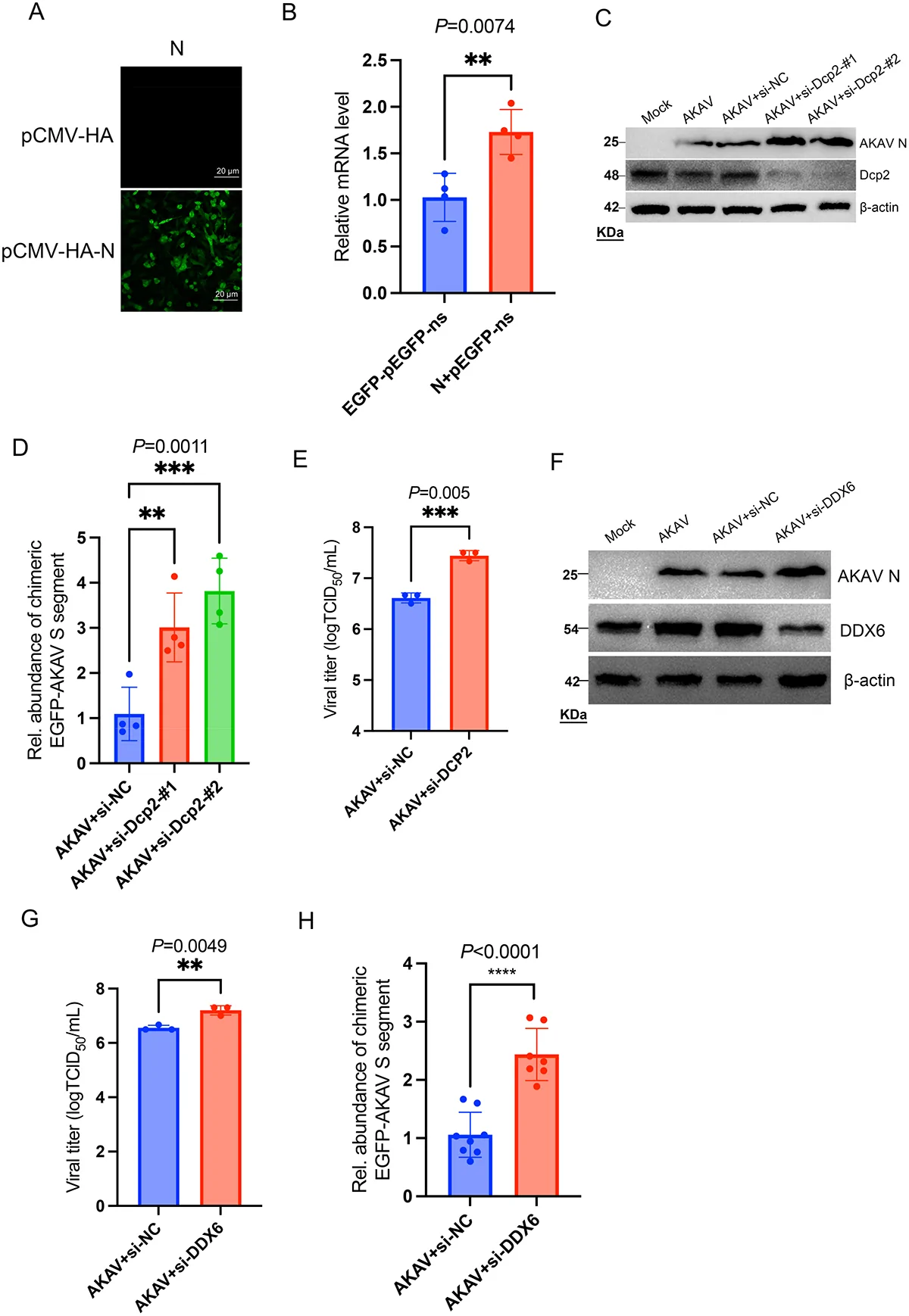
PBs inhibition facilitates virus replication.

(A) BHK-21 cells were transfected with pCMV-HA-N or pCMV-HA for 36 h, and IFA was applied to detect the expression of N protein. (B) The effect of N protein overexpression on mRNA stability was detected by qRT-PCR. (C) BHK-21 cells were transfected with siRNAs targeting Dcp2 for 24 h, then infected with AKAV at 1 MOI, and Western blot was applied to detect the expression of viral N protein and Dcp2. (D) BHK-21 cells were transfected with Dcp2-targeting siRNAs for 24 h, followed by 24 h of AKAV infection. Then the viral titers were subsequently quantified via TCID_50_. (E) BHK-21 cells were transfected with Dcp2-targeting siRNAs and pEGFP-ns for 24 h, followed by 12 h of AKAV infection. The total RNA was extracted and subjected to qRT-PCR analysis. (F) BHK-21 cells were transfected with siRNAs targeting DDX6 for 24 h, then infected with AKAV at 1 MOI, and Western blot was applied to detect the expression of viral N protein and DDX6. (G, H) Effects of DDX6 silencing on viral replication and cap-snatching efficiency were determined as described above.

Previous reports showed that depletion of DDX6 inhibits PBs formation [25]. To investigate the effects of PBs on AKAV replication, we then blocked PBs assembly by silencing DDX6. We found that DDX6 depletion markedly promoted virus replication (Figure 9(F,G)). Furthermore, the cap-snatching efficacy of AKAV increased by 2.5-fold following DDX6 knockdown (Figure 9(H)). Taken together, AKAV-mediated PBs disassembly prevents mRNA degradation and may supply additional mRNA caps to facilitate viral transcription and subsequent replication.

## Discussion

AKAV belongs to the genus *Orthobunyavirus*. It can cause severe reproductive failures in sheep and cows [4]. To complete its life cycle, AKAV has to transcribe viral mRNAs by snatching caps from cellular mRNAs. However, how AKAV optimizes this process remains unclear. In this study, we report that AKAV infection disassembles polysomes, SGs, and PBs to liberate mRNAs and promote the cap-snatching process (Figure 10).

**Figure 10.**
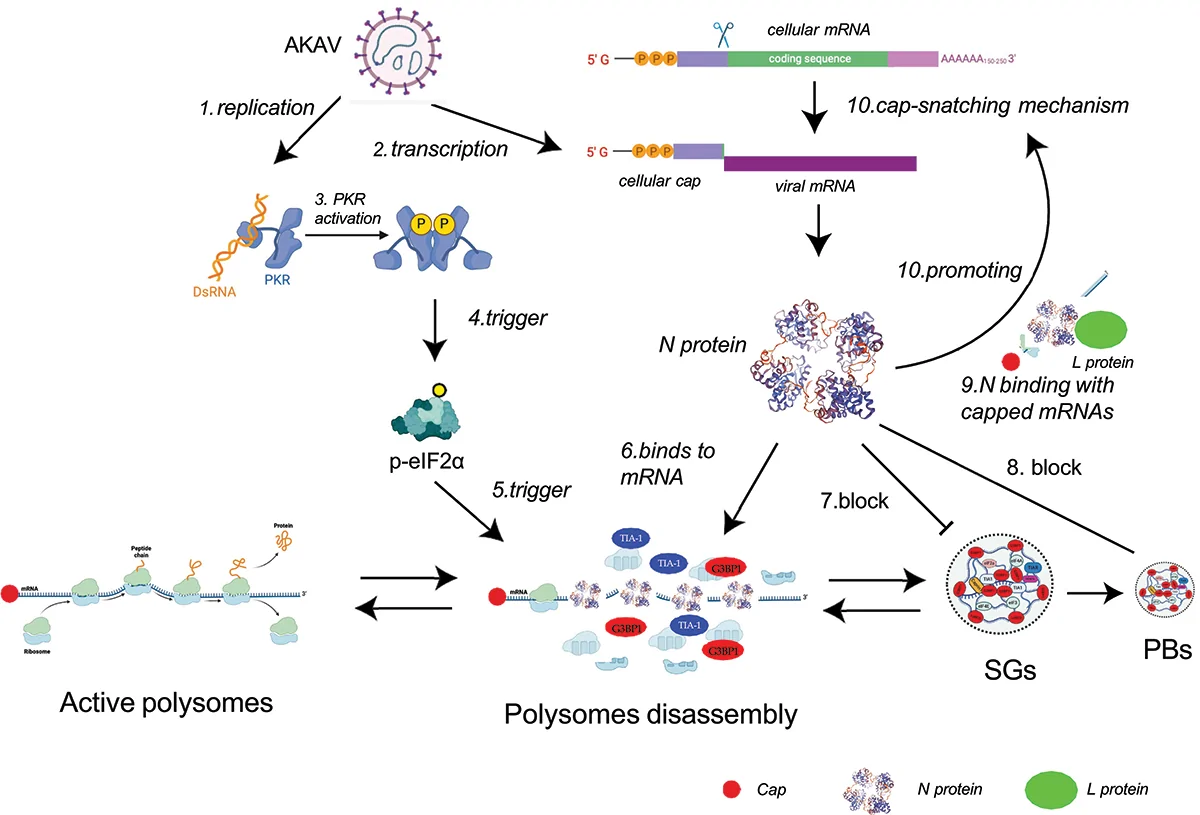
The schematic diagram of AKAV reprogramming the PKR -eIF2α-SGs/PBs axis to promote cap-snatching.

AKAV infection activates PKR, induces eIF2α phosphorylation, and subsequently disassembles polysomes to liberate intracellular mRNAs. The viral N protein then competes with host RNA-binding proteins for mRNA binding and disrupts SGs/PBs assembly, thereby creating an optimal cellular environment for viral cap-snatching.

Unlike the influenza virus, which snatches caps in the nucleus, AKAV initiates genome transcription via cap-snatching in the cytoplasm using mRNAs as cap sources [26]. It is hypothesized that there may be a viral protein that functions as a “cap trapper” similar to PB2 of the influenza virus during the cap-snatching process of bunyaviruses [27]. A cap-binding function has been hypothesized in the N protein of arenavirus and Sin Nombre virus (SNV) [17,28–30]. However, there is insufficient evidence to confirm the existence of the proposed cap-binding site, whereas the L could interact with the N protein and has been reported to possess cap-binding capacity [31,32]. The L protein, N protein, and mRNAs may form a trimer to facilitate the cap-snatching process.

mRNAs are typically distributed in polysomes, SGs, and PBs. Being the primary cap sources for AKAV transcription, cellular mRNA existence forms are critical. As cellular mRNAs serve as the primary cap donors for AKAV transcription, their subcellular distribution is critical. It is reported that SGs and PBs are involved in cap-snatching for hantaviruses and tomato spotted wilt virus [16,17]. Surprisingly, SGs and PBs are largely disassembled during AKAV infection, and the N protein mediates both of these processes via binding to mRNAs, according to our study. It is worth noting that, during the early stage of infection, several PBs dock onto the N protein foci, and as the infection progresses, these PBs vanish. This discrepancy is probably attributed to differences in the N protein structure and affinity among these viruses. We hold that SGs and PBs are not the cap-snatching sites of AKAV for the following reasons. Firstly, it is evolutionarily disadvantageous for viruses to hinder the machineries required for their own replication. Secondly, SGs and PBs inhibition promotes cap-snatching efficacy and replication, rather than the reverse. Thirdly, mRNAs localized in SGs and PBs are wrapped by various RNA-binding proteins, which may form steric hindrance to inhibit the L protein-mediated cap-snatching process. Fourthly, some bunyaviruses’ replication is not dependent on PBs assembly [16]. Disruption of PBs by DDX6 silencing leads to enhanced AKAV replication and improved cap-snatching efficacy. Additionally, N protein-mediated PBs assembly prevents mRNAs from degradation, which may provide more caps for cap-snatching. It should be noted that mRNAs targeted to PBs are more frequently utilized for cap-snatching than translating mRNAs, possibly because polysome-wrapped mRNAs are inaccessible to viral N and L proteins. Collectively, PBs may not be the cap-snatching sites; instead, the mRNAs sequestered in PBs could serve as the cap source for AKAV.

As for mRNAs bound to polysomes, they exhibit significantly higher affinity than other cytosolic RNA-binding proteins, such as G3BP1, DDX6, and even N protein [6]. To overcome this barrier, AKAV activates the PKR-eIF2α axis to block translation initiation and subsequent polysome disassembly, thereby exposing mRNAs to the viral N protein for cap-snatching. Interestingly, eIF2α phosphorylation did not influence the translation of viral proteins, indicating that AKAV has evolved a mechanism to bypass the typical eIF2α-dependent regulation of translation. Unlike sandfly fever Sicilian virus, its nonstructural protein NSs binds to eIF2B to prevent its inactivation [33]. Notably, mRNAs targeted for PBs are more frequently utilized in cap-snatching than translating mRNAs, possibly because polysome-wrapped mRNAs are inaccessible to N and L proteins. However, the binding affinity of N-mRNAs and G3BP1/DDX6-mRNA is not analyzed in this study.

Activation of PKR in virus-infected cells may be mediated by viral double-stranded or structured single-stranded RNAs with a 5’-triphosphate cap [34,35]. PKR activation is tightly regulated by cellular factors such as PACT, which prevents its aberrant activation by self-derived dsRNAs [36]. During AKAV replication, newly synthesized viral RNAs are encapsidated by the N protein; as observed in AKAV-infected cells, this encapsidation prevents the formation of dsRNA replication intermediates. However, the mechanisms underlying PKR activation (in this context) remain to be elucidated. Furthermore, whether PKR activation has direct or indirect promoting effects on AKAV replication through other pathways still requires further investigation.

To verify the interaction between N and mRNAs, we investigated the localization between them. First, cells were labeled with Cy5-linked oligo-dT to target mRNAs, and IFA results showed N colocalized with poly(A)-containing RNA. Subsequently, we used an antibody against PABP1 to label the mRNAs in the cytosol. It was reported that the release of PABP1 from polysomes is necessary for its nuclear import [37,38]. In AKAV-infected cells, PABP1 was predominantly distributed in the nucleus, implying the polysomes were disassembled during virus infection. Early in infection, N colocalized with granule-like PABP1 (a protein recruited to SGs upon cellular stress), indicating SG assembly was initially followed by disassembly [39]. N forms a tetramer, and each N subunit binds to four nucleic acids in its RNA-binding slot. These results suggested that the N protein might bind to the mRNAs and subsequently release PABP1, leading to its redistribution. Mutation of the N protein’s RNA-binding amino acids abolishes its ability to inhibit SGs and PBs formation.

In the protein competition assay, our findings indicated that AKAV infection was capable of inhibiting the association between G3BP1 and mRNA. This inhibitory effect persisted even when the samples were treated with AS, but the extent to which the inhibitory effect of the N protein is attenuated after mutation of the amino acids at the RNA-binding site still requires further investigation. We also found that overexpression of the N protein led to the disassembly of SGs and PBs. These findings suggest that the viral N protein likely competes with specific RNA-binding proteins (RBPs; e.g. G3BP1 and DDX6) for mRNA binding or by the broader remodeling of cytoplasmic mRNP complexes during infection, thereby mediating the disassembly of SGs and PBs, even though AKAV activates the upstream PKR-eIF2α signaling pathway. Furthermore, SGs are generally known as signaling hubs to boost innate immune responses to promote the elimination of viral infections [8,40]. Future investigations are required to comprehensively probe the intricate interplay between SGs and the innate immune response.

Many questions remain. First, we did not determine whether the N protein preferentially binds to mRNA or other RNA species. Second, rescue of the mutant AKAV was unsuccessful, and we did not assess the cap-snatching efficiency or *in vivo* virulence of AKAV mutants deficient in SG and PB inhibitory activity. Third, it remains unclear whether capped mRNAs exhibit a binding preference, and the subcellular location of cap-snatching has not yet been determined. Fourth, the interaction between AKAV and SGs/PBs at the early stage of infection still requires further elucidation.

In our research, we discovered novel functions of the N protein and SGs during the cap-snatching process of the AKAV. These findings significantly enhance our understanding of the AKAV cap-snatching step and also elucidate how the virus hijacks cellular machinery to complete its lifecycle. Moreover, the results offer valuable insights into the cap-snatching mechanisms of other negative-sense, cytoplasm-replicating RNA viruses.

## Experimental procedures

### Cell lines, virus, antibodies, and chemicals

BHK-21, Vero, and bovine trophoblast cells (BTCs) were all cultured in Dulbecco’s modified Eagle’s medium (DMEM; Invitrogen, Carlsbad, CA, USA) containing 10% (v/v) fetal bovine serum (FBS) in a humidified 37 °C, 5% CO_2_ incubator. The AKAV strain TJ2016 (GenBank accession numbers: MT761689.1, MT761688.1, and MT755621.1) used in this study was stored in our lab. To inactivate AKAV, 1 mL of virus stock was diluted to 1 MOI in a 60 mm cell culture dish (Corning, USA) and irradiated by the UV source (low-pressure mercury vapor discharge lamp) for 4 h. Antibodies and chemicals used in this study are listed in Table 1.

**Table 1.** Antibodies and chemicals used in this study.

Antibody/chemical	Source	Product code
Anti-G3BP antibody [EPR13986(B)]	Abcam	ab181150
DDX6 Rabbit pAb	ABclonal	A16270
SCICONS anti-dsRNA antibody (J2)	Nordic-Mubio	10,010,200
Biotinylated Oligo(dT) Probe (50pmol/µl)	Promega	Z5261
HA-Tag (6E2) Mouse mAb	Cell Signaling	2367
Mouse Anti-β actin mAb	ZSBIO	TA-09
HRP labeled goat anti rabbit IgG (H+L)	ZSBIO	ZB-2301
HRP labeled goat anti mouse IgG (H+L)	ZSBIO	ZB-2305
Anti-GRP78 BiP antibody	Abcam	ab21685
Phospho-eIF2α (Ser51) (D9G8) Rabbit mAb	Cell Signaling	3398
eIF2α Antibody	Cell Signaling	9722
Anti-PKR (phospho T446) antibody [E120]	Abcam	ab32036
Anti-PKR antibody	Abcam	ab32052
PERK/EIF2AK3 Polyclonal antibody	Proteintech	24,390
Phospho-PERK/EIF2AK3 (Ser719) Polyclonal antibody	Proteintech	29,546
Vari Fluor 488-Streptavidin	MedChemExpress	HY-D1808
Goat anti-Mouse IgG (H+L) Cross-Adsorbed Secondary Antibody, Alexa Fluor™ 488	Invitrogen	A-11001
Goat anti-Rabbit IgG (H+L) Cross-Adsorbed Secondary Antibody, Alexa Fluor™ 568	Invitrogen	A-11011
SuperStar Universal SYBR Master Mix	CWBIO	CW3360M
FastKing RT Kit (With gDNase)	TIANGEN	KR116
ClonExpress II One Step Cloning Kit	Vazyme	C112
Trans DNA Maker I	Transgene	BM401
Trans2K Plus II DNA Marker	Transgene	BM121
Western and IP cell lysis (without inhibitors)	Solarbio	R0100
PMSF (100 mM)	Beyotime	ST506
10X Phosphatase inhibitor complex III	Sangon Biotech	C500019
Immobilon-PSQ membrane, PVDF, 0.2 µm	Millipore	ISEQ00010
5X SDS-PAGE Loading Buffer (Reduced)	Affinibody	AIWB0025
PAGE Gel One-Step Preparation Kit	Affinibody	NSF12
Robust ECL Solution	Affinibody	AIWB-006
Tri-Color Prestained Protein Marker	Affinibody	AIWB-011plus
Circle Microsope Cover Glass	NEST	801,009
DAPl solution(ready-to-use)	Solarbio	C0065
Aqua-Poly/Mount	Polsciences	18,606
Dulbecco’s Modified Eagle Medium (DMEM)	Gibco	12,100,046
FBS	Gibco	10099141C
PKR-IN-C16	MedChemExpress	HY-13977A
DL-dithiothreitol	MedChemExpress	HY-15917
LIPOFECTAMINE™ 3000 Transfection Kit	Invitrogen	L3000075
OPTI-MEM(R) I Reduced Serum Medium (1X)	Gibco	3,048,024
Mouse anti-AKAV N 2D3 antibody	Prepared in this study	
Rabbit anti-AKAV N Polyclonal antibody	Prepared in this study	
Mouse anti-SBV N antibody	Prepared in this study	

### Plasmid construction

The genes encoding AKAV proteins were amplified by PCR and cloned into the pCMV-HA vector via homologous recombination. The genes of the N proteins from Oropouche virus (OROV) (GenBank no: KP691605.1), Hantavirus (GenBank no: NC_005218.1), and Bunyamwera virus (BUNV) (GenBank no: NC_001927.1) were synthesized by Beijing Tsingke Biotech Co., Ltd.

The cap-donor plasmid pEGFP-ns was constructed based on pEGFP-N2 by homologous recombination with primers pEGFP-ns-F/R to prematurely terminate EGFP expression by introducing a two-nucleotide frameshift mutation (Figure 5(C)). All primers used in this study are listed in Table 2.

**Table 2.** Primers used in this study.

Primer name	Primer sequence	Application
pCMV-HA-F	ATTCGGTCGACCGAGATCTCT	pCMV-HA linearization
pCMV-HA-R	CATGGCCATAAGAGCGTAATCTG	
AKAV-N-F	ATTACGCTCTTATGGCCATGATGGCAAATCAATTCATTTTCAAC	pCMV-HA-N construction
AKAV-N-R	GAGATCTCGGTCGACCGAATTTAGATCTGGATACCAAATTGAGCC	
K56A-F	TCTTACATGCGACGCCACAACCAAGTGTCGAT	pCMV-HA-N mutant construction
K56A-R	TGGCGTCGCATGTAAGACCATCTTGGCCTTCTT	
H94A-R95A-F	TTACGCTCGCGGCCATCTCGGGCTACTTAGCTCGC	
H94A-R95A-R	AGATGGCCGCGAGCGTAAAGGCAGTGTCTGACA	
AKAV-Nss-F	ATTACGCTCTTATGGCCATGATGTTCCACAACGGAATGCAG	pCMV-HA-Nss construction
AKAV-Nss-R	GAGATCTCGGTCGACCGAATCTAAGTAGCCCGAGATGCGATG	
AKAV-G2/Gn-F	ATTACGCTCTTATGGCCATGATGATTATTACAATTCTCAACGTGCTT	pCMV-HA-Gn construction
AKAV-G2/Gn-R	GAGATCTCGGTCGACCGAATTTACCTCGTTTTAGTCAGGGTTTTG	
AKAV-Nsm-F	ATTACGCTCTTATGGCCATGTATTTATGTAAAAAAAGACTCCCCAATAT	pCMV-HA-Nsm construction
AKAV-Nsm-R	GAGATCTCGGTCGACCGAATTTAGGATGATTTAGTTATCAGATAATCGAA	
AKAV-G1/Gc-F	ATTACGCTCTTATGGCCATGACATCTAAAGATCTTCATAGTGCTACTATTCA	pCMV-HA-Gc construction
AKAV-G1/Gc-R	GAGATCTCGGTCGACCGAATTTATTTCAGCTTATTTTCTAGTTTGTACACT	
AKAV-L-HA-F	CCATACGATGTTCCAGATTACGCTTAAATGAGTTGATAGAGATCAGTACGATTT	pCMV-HA-L construction
AKAV-L-HA-R	TCTGGAACATCGTATGGGTAAAAATCAAATTTTGATCGACCACC	
SBV-N-F	ATTACGCTCTTATGGCCATGATGTCAAGCCAATTCATTTTTGAA	pCMV-HA-SBV N construction
SBV-N-R	GAGATCTCGGTCGACCGAATTTAGATGTTGATACCGAATTGCTGC	
OROV-N-F	ATTACGCTCTTATGGCCATGTCCGAGTTCATCTTCAACGATG	pCMV-HA-OROV N construction
OROV-N-R	GAGATCTCGGTCGACCGAATTCAAATATCGATACCGAACTGTCTCA	
pEGFP-ns-F	GGGCGAGTAAAGCTGTTCACCGGGGTGGTGCC	pEGFP-ns construction
pEGFP-ns-R	AACAGCTTTACTCGCCCTTGCTCACCATGGTG	
Cap-N-F1	ACCGTCAGATCCGCTAG	Chimeric Viral-EGFP-ns RNA detection
Cap-N-F2	GTCAGATCCGCTAGCGC	
Cap-N-F3	TCAGATCCGCTAGCGCTAC	
Cap-S-R	GGAAATGGTTATTAACCACTG	

### Transfection

Cell transfection was performed following the manufacturer’s instructions for Lipofectamine™ 3000 reagent (Thermo Fisher Scientific, Cat. No. L3000015). Briefly, cells were seeded in 6-well plates. When cell confluence reached 60–80%, Lipofectamine™ 3000 reagent and plasmids were separately diluted in Opti-MEM medium. The diluted Lipofectamine™ 3000 was gently mixed with DNA, and the mixture was incubated at room temperature (RT) for 10 min. The mixture was then added dropwise to the cell culture medium, followed by gentle plate agitation. Cells were further cultured in an incubator for 36 h before subsequent experiments.

### Western blot

Cells were rinsed with phosphate-buffered saline (PBS) and lysed in ice-cold RIPA buffer (25 mM Tris-HCl, pH 7.6, 150 mM NaCl, 1% NP-40, 1% sodium deoxycholate, and 0.1% SDS) containing PMSF (Beyotime, ST506) and a phosphatase inhibitor (Sangon, C500019) for 10 min on ice. Lysates were centrifuged, and the supernatant was mixed with 5× SDS loading buffer and boiled for 10 min. The samples were separated by 12% SDS-PAGE and transferred to PVDF membranes. Membranes were blocked with 5% bovine serum albumin (BSA) in PBS and probed with primary antibodies overnight at 4°C. Following three washes with PBST, membranes were incubated with HRP-conjugated secondary antibodies. Signal detection was performed using a chemiluminescent substrate (Affinibody, AIWB-006) and visualized with an Odyssey Fc imaging system (LI-COR). Band intensities were quantified using ImageJ software.

### Immunofluorescence assay (IFA)

Cells were seeded on coverslips in 6-well plates. At 60%–80% confluence, the cells were infected with AKAV. Before harvesting, SGs were induced by treating cells with either 500 mM AS (sodium arsenite) or 2 μM DTT (dithiothreitol) for 30 min. Cells were then fixed with 3.7% paraformaldehyde in PBS for 10 min at RT, washed three times with PBS (5 min each), permeabilized with 0.1% Triton X-100 in PBS for 10 min, and blocked with 1% BSA in PBS for 30 min at RT. Primary antibodies were added and incubated overnight at 4°C. After three PBS washes, Alexa Fluor 488- or 594-conjugated secondary antibodies (Thermo Fisher) were applied and incubated for 1 h at RT. Nuclei were stained with DAPI (Sigma-Aldrich) for 8 min. Finally, cells were imaged using a Leica Stellaris 5 confocal microscope, and images were processed with ImageJ software (Version 2.14.0).

### Translation efficacy assay

To measure protein synthesis rates, puromycin incorporation efficiency was analyzed as previously described [22]. Briefly, before harvesting, Vero or BHK-21 cells were incubated with 0.5 mM puromycin at 37°C for 25 min. Subsequently, protein extracts were prepared, and SDS-PAGE and western blotting were conducted to assess puromycin incorporation. Moreover, an IFA was performed using primary antibodies against puromycin and N proteins.

### Co-IP

BHK-21 cells infected with AKAV for 24 h were lysed using a cell lysis buffer (Solarbio Life Science, R0100, Beijing) containing 1 mM PMSF (Beyotime Biotechnology, Beijing) and 1× protease inhibitor mixture (MedChemExpress, Shanghai). First, wash Protein A/G agarose beads three times with IP lysis buffer. Then, add the washed beads to the cell lysate supernatant and incubate at 4°C for 60 min with mixing. After centrifugation, collect the supernatant and add the antibody against the target protein, followed by a 4-h incubation at 4°C. Next, add the prewashed Protein A/G agarose beads again and incubate at 4°C for 2 h with mixing. After centrifugation and discarding the supernatant, wash the agarose beads three times with prechilled IP lysis buffer. Finally, perform a Western blot on the samples to detect the target protein.

### Poly(A) RNA Co-IP

The Poly(A) RNA Co-IP assay was performed following a previously described protocol^12^. Briefly, BHK-21 cells were either infected with AKAV at an MOI of 5 for 24 h or treated with 500 μM AS for 1 h at 37°C. After treatment, cells were washed three times with PBS and collected in 200 μL of lysis buffer (20 mM Tris-HCl, pH 7.5, 2.5 mM MgCl_2_, 100 mM NaCl, 0.5% Triton, protease inhibitors, and 0.2 U/mL RNase inhibitors). The cell suspension was incubated on ice for 20 min and centrifuged at 1,000 × g for 5 min at 4°C. To the supernatant, 100 μL of oligo-dT Dynabeads (Invitrogen), pre-equilibrated with lysis buffer, were added, and the binding reaction was conducted for 30 min at 4°C on a rotating wheel. The Dynabeads were then washed five times with ice-cold buffer (20 mM Tris-HCl, pH 8, 150 mM NaCl, 0.02% NP−40, and 0.1 U/mL RNase inhibitors) for 3 min each. Next, 50 μL of 20 mM Tris-HCl (pH 7.5) was added to the beads, followed by heating at 80°C for 45 s. The supernatant was quickly separated from the beads. Half of the sample underwent Western blot analysis using primary antibodies against N and G3BP1. As a negative control, the entire experiment was repeated with the addition of 0.1 U/mL RNase A (Invitrogen).

### Analysis of cap-snatching efficiency

First, the pEGFP-ns plasmid was generated by introducing a two-nucleotide frameshift mutation within the multiple cloning site, which is designed for transcribing nonsense mRNAs and used as “cap donors.” When BHK-21 cells reached ~70% confluency, they were transfected with pEGFP-ns for 12 h, then infected with AKAV at an MOI of 1 and treated with 10 nM PKR-IN-C16, followed by harvesting 12 h later. Total RNA was extracted using TRIzol, reverse-transcribed into cDNA, and analyzed by qPCR.

### Quantitative reverse transcription polymerase chain reaction (RT-qPCR)

Cells were homogenized in TRIzol Reagent, followed by RNA extraction by chloroform-mediated phase separation. The RNA was precipitated with isopropanol, washed with ethanol, and then dissolved in DEPC-treated water. cDNA was reverse-transcribed from purified RNA using the FastKing RT Kit (TIANGEN, KR116). qPCR reaction mixtures were prepared with SYBR Green PCR Master Mix (CWBIO, CW3360), specific primers (Table 1), and cDNA. Amplification was performed on the StepOne™ Real-Time PCR System under optimized cycling conditions. Data were analyzed using StepOne™ Software.

### Polysome profiling

Polysome profiling was performed using an established protocol with modifications provided by Hangzhou NeoRibo Biotechnology Co., Ltd. Briefly, BHK-21 cells were infected with AKAV at an MOI of 5 for 12 h, rinsed twice with PBS, harvested, and stored at −80°C until use. Cells were lysed under conditions that preserve polysomes, and the clarified lysates were layered onto a 10–50% (w/v) linear sucrose gradient. Following ultracentrifugation in an SW41 rotor at 38,000 rpm for 3 h at 4°C, the gradients were fractionated using a density gradient fractionation system with continuous monitoring of absorbance at 254 nm.

### Statistical analysis

All data were processed with GraphPad Prism 6 (GraphPad Software Inc.). A Student’s t‑test or nonparametric test was used to analyze the difference between the values of two groups. A value of *p* < 0.05 was considered statistically significant.

## Supplementary Material

_Revised_Supporting_Information_clean.docx

## Data Availability

All data are contained within the manuscript, and the raw data are openly available via the following DOI:10.5281/zenodo.22796626.

## Abbreviations

AKAV: Akabane virus
SGs: stress granules
PBs: processing bodies
NMD: nonsense-mediated decay
RBPs: RNA-binding proteins
PKR: double-stranded RNA-dependent protein kinase
G3BP1: Ras GTPase-activating protein-binding protein 1
Dcp2: decapping protein 2
DDX6: DEAD-box helicase 6
PABP1: Poly A binding protein 1
RdRp: RNA-dependent RNA polymerase
ISG: interferon-stimulated gene
PMSF: phenylmethylsulfonyl fluoride
PKR-IN-C16: PKR Inhibitor C16
