# Supplementary material for: Akabane virus reprograms the PKR‑eIF2α‑SGs/PBs axis to fine-tune cap-snatching efficiency
Source: Virulence. 2026 Oct 12;17(1):2748149. doi: 10.1080/21505594.2026.2748149 (PMC13679326; doi:10.1080/21505594.2026.2748149)
Supplement: Supplementary file 1 [file KVIR_A_2748149_SM8583.docx]

**Supporting Figure Legends**

**Fig. S1 AKAV infection inhibits SGs formation in Vero and BTCs**

(A) BTCs were infected with AKAV, and subjected to IFA at 12, 18, and 24 hpi as described above. (B) AKAV-inoculated BTCs were treated with AS before being subjected to IFA. (C) The formation of SGs in AKAV-infected Vero cells was detected by IFA. (D) The formation of SGs in AKAV-infected Vero cells following treatment with AS or DTT was examined by IFA. (E) Statistical analysis of SG-positive cells was performed by counting cells in 3 random fields per group, including AKAV-infected (24 h), AS-induced, DTT-induced, and mock-infected groups. The data were presented as mean ± SD (t-test, * indicates p < 0.01). Scales bar=10 μm.

**Fig. S2 AKAV infection triggers the phosphorylation of eIF2α via PKR in Vero cells**

(A) Vero cells were infected with AKAV. At indicate timepoints, the cells were collected and subjected to western blot to detect the expression and phosphorylation of eIF2α. (B) The expression and phosphorylation of PKR and the G3BP1 status were detected by Western blotting in AKAV-infected Vero cells. (C) The puromycin incorporation was analyzed by IFA in AKAV-infected BHK-21 cells. (D) AKAV infection inhibits SGs formation. BHK-21 cells were infected with AKAV. At 12, 18, and 24 hpi, cells were fixed for IFA. Anti-eIF4G antibody (red) was used to label SGs. As a positive control, cells were treated with DTT (2 μM, 1 h) prior to IFA. (E) AKAV infection induces PKR and eIF2α phosphorylation. BHK-21 cells were infected with AKAV at an MOI of 5. Whole-cell lysates were harvested at 1, 2, 3, and 4 hpi for Western blotting.

**Fig. S3** (A)The alignment of N protein sequences between AKAV, BUNV, SBV, and OROV by Jalview software. (B) AKAV N protein dose not affects eIF2α phosphorylation. BHK-21 cells were transfected with pCMV-HA-N for 36 h, and whole-cell lysates were harvested and subjected to Western blot analysis. (C) PT129 promotes AKAV N protein expression. BHK-21 cells were infected with AKAV at an MOI of 0.1, and whole-cell lysates were harvested at 12 and 24 hpi for Western blot analysis.

**Fig. S4 The distribution of N protein and** **poly (A)-containing RNA.**

BHK-21 cells were infected with AKAV for 24 h, followed by IFA to detect viral N protein and poly(A)-containing RNA.

**Fig. S5** (A) Screening of primer sets for chimeric RNA detection; (B) SGs inhibition promotes AKAV cap-snatching. BHK-21 cells were pretreated with PT129 (5μM) for 12 h prior to AKAV infection at an MOI of 5 for 4 h, and cap-snatching efficiency was determined using qRT-PCR.

**Fig. S6 N protein expression inhibits PBs assembly in Vero cells**

Vero cells were transfected with pCMV-N for 24 h, followed by IFA to detect viral N protein and PBs marker DDX6.
